# Proliferation and benevolence—A framework for dissecting the mechanisms of microbial virulence and health promotion

**DOI:** 10.1111/eva.12952

**Published:** 2020-03-31

**Authors:** Kristofer Wollein Waldetoft, Lars Råberg, Rolf Lood

**Affiliations:** ^1^ School of Biological Sciences Georgia Institute of Technology Atlanta GA USA; ^2^ Department of Biology Lund University Lund Sweden; ^3^ Division of Infection Medicine Department of Clinical Sciences Lund University Lund Sweden

**Keywords:** commensal, evolution, infection, probiotic, resistance, symbiosis, tolerance, virulence, virulence factor

## Abstract

Key topics in the study of host–microbe interactions—such as the prevention of drug resistance and the exploitation of beneficial effects of bacteria—would benefit from concerted efforts with both mechanistic and evolutionary approaches. But due to differences in intellectual traditions, insights gained in one field rarely benefit the other. Here, we develop a conceptual and analytical framework for the integrated study of host–microbe interactions. This framework partitions the health effects of microbes and the effector molecules they produce into components with different evolutionary implications. It thereby facilitates the prediction of evolutionary responses to inhibition and exploitation of specific molecular mechanisms.

## 1 INTRODUCTION

Microbes have profound effects on the health of their hosts, and host–microbe interactions are therefore subject to intense research from both mechanistic and evolutionary perspectives. Traditionally pursued in isolation, these lines of inquiry are now becoming increasingly intertwined (Bordenstein & Theis, 2015). This is especially so in the work on antibiotic resistance evolution, and the consequent search for molecular targets for evolution‐proof drugs, including antivirulence therapeutics (Allen, Popat, Diggle, & Brown, 2014). Progress is hampered, however, by a lack of common conceptual ground; key concepts in one field, such as “virulence factor” in microbial pathogenesis research (Falkow, 1988, 2004) do not fit into the conceptual structure of the other, for example the trade‐off paradigm in virulence evolution theory (Alizon, Hurford, Mideo, & Van Baalen, 2009) (because the former is focused on the mechanisms of host harm, whereas the latter disregards mechanism, and assumes relationships between host harm and pathogen fitness). This is unfortunate because pressing public health challenges, most notably antibiotic resistance, are complex and have several aspects that are studied in both fields.

The aim of this paper is to lay a foundation for a common conceptual framework for host–microbe interactions, in which mechanistic and evolutionary traditions can be integrated. To this end, we combine the analytical approach of resistance–tolerance theory in evolutionary ecology (Råberg, Sim, & Read, 2007) with the experimental strategy for identifying virulence factors in microbial pathogenesis research (Falkow, 1988). Whilst most commonly applied to pathogens in acute infections, this type of experiment can identify a range of molecules that impact host health, whether the effect is detrimental or beneficial, the microbe obligate or opportunistic, and the condition acute or chronic. And we intend our analysis to be equally broad. The framework we propose is visual and intuitive, but it also has a simple statistical formalization based on generalized linear models, which makes it flexible and open for further developments. In the main text, we develop the concepts and their implications under the assumption that the microbe's health effect and fitness are linearly related to its proliferation in the host. This makes practical sense and is consistent with the results of a recent meta‐analysis (Acevedo, Dillemuth, Flick, Faldyn, & Elderd, 2019). In Box 1, we provide formal analyses and extend these beyond the linear case.

Box 1 **Statistical approaches to testing for variation in resistance and tolerance, and proliferation and benevolence**

If the focus of the study is resistance and tolerance, two or more host types should be infected by a given type of microbe, or, if the focus is a particular molecular factor encoded by the host, a wild‐type host and its isogenic factor‐negative knockout should be used. To test for variation in resistance, an analysis (e.g., *t* test or ANOVA) with microbial density against host type is performed (density = host type), where a significant effect of host type indicates variation in resistance. To test for variation in tolerance, an analysis (e.g., ANCOVA) with host health against host type, bacterial density, and their interaction, is performed (health = host type + density +density × host type). Here, a significant interaction between microbial density and host type (density × host type) indicates that the slope of the relationship between host health and microbial density varies among host types, that is, there is variation in tolerance. If a wild‐type host has a higher resistance (lower microbial density) than its isogenic factor‐negative knockout, the factor is a resistance factor, and if it has a greater tolerance (shallower slope of health on density), the factor is a tolerance factor.

If the focus of the study is instead proliferation and benevolence, a single host type should be infected by two or more strains of a microbe, or, if the focus is a particular molecular factor encoded by the microbe, a wild type strain and its isogenic factor‐negative knockout. The same approach as above can then be used to test for variation in proliferation and benevolence among bacterial strains. Thus, a significant effect of strain in a model (e.g., *t* test or ANOVA) with density against strain (density = strain) would indicate variation in proliferation. A significant interaction between strain and density (strain × density) in a model (e.g., ANCOVA) with health against strain, density, and their interaction (health = strain +density + strain × density) would indicate variation in benevolence. If a wild type microbe has a higher proliferation (higher microbial density) than its isogenic factor‐negative knockout, the factor is a proliferation factor, if it has a higher benevolence (more positive or less negative slope of health on density), the factor is a benevolence factor, and if it has a lower benevolence (less positive or more negative slope), that factor is a malevolence factor.

In case the data are non‐normally distributed, for example if the outcome of infection is measured in terms of survival (0 or 1) instead of a quantitative health measure, a generalized linear model with appropriate error distribution (binomial in case of survival) should be used instead of ANCOVA.

In principle, it is possible to combine the two approaches in one experiment, and infect ≥2 host types with ≥2 microbial strains in a fully factorial design. A model with density against host type and strain (density = host type + strain) would test for variation in resistance and proliferation. A model with health against density, host type, strain, and their interactions with density (health = host type + strain + density + host type × density + strain × density) would test for variation in tolerance and benevolence. In such a combined experiment, it is advisable to initially also include the interaction between host type and strain. If nonsignificant, this term may be removed. If significant, it would indicate that the outcome depends on the specific combination of host type and strain.

As always, it is important to scrutinize the data before analysis to make sure that model assumptions are fulfilled. In particular, it is key to check that there is indeed a linear relationship between health and density. In case of nonlinearity, the model can be modified to fit the shape of the relationship between health and density, for example by including a quadratic term (density^2^) and its interaction with strain, as has previously been done in analyses of tolerance (Regoes et al., 2014).

If the aim is to obtain a precise estimate of the heritability of benevolence or proliferation (rather than just test for the presence of genetic variation in these traits), analyses can be performed with methods that take relatedness among strains into account (see Hodcroft et al., 2014).

Moreover, in analyses of tolerance and benevolence it is important to recognize that limited overlap in microbial density between host types/strains can result in spurious effects (see Råberg, Graham, & Read, 2009).

In the following, we first discuss contemporary matters at the crossroads of mechanistic and evolutionary research, to illustrate the problem. We then introduce the two research traditions on which we build: the mechanistic study of microbial health effects and the evolutionary analysis of host resistance versus tolerance to infection. On this basis, we propose the proliferation–benevolence framework. The paper finishes with a review of empirical evidence for these two components—proliferation and benevolence—of microbial health effects.

## 2 RESEARCH AT THE CROSSROADS

### 2.1 Virulence factor inhibitors as evolutionarily robust therapeutics

Antibiotic resistance is a major public health challenge. It evolves because antibiotics kill bacteria, and the widespread use of these drugs thus imposes strong selection on the bacteria to survive in their presence. On this basis, a view has emerged that a more evolutionarily robust approach would be to target virulence factors—the microbial molecules responsible for pathogenesis—in order to “disarm rather than kill” the pathogen (Weigert et al., 2017), and there is a growing literature on different aspects of this approach as well as the potential consequences of its implementation (Allen et al., 2014; Brown, Cornforth, & Mideo, 2012; Cegelski, Marshall, Eldridge, & Hultgren, 2008; Defoirdt, 2016; Vale et al., 2016; Wollein Waldetoft & Brown, 2017).

However, virulence factors can harm the host in different ways. As a concrete example, *Staphylococcus aureus* and *Streptococcus pyogenes* produce a number of proteins (e.g., the streptococcal antiphagocytic M protein) that protect the bacteria from immune‐mediated killing (Åkesson, Sjöholm, & Björck, 1996; Carlsson, Sandin, & Lindahl, 2005; Dossett, Kronvall, Williams, & Quie, 1969; Horstmann, Sievertsen, Knobloch, & Fischetti, 1988). These are virulence factors, because they contribute to host harm, but their inactivation results in increased killing of the bacteria (Courtney, Hasty, & Dale, 2006; Falugi, Kim, Missiakas, & Schneewind, 2013; Frick, Åkesson, Rasmussen, Schmidtchen, & Björck, 2003). The expected evolutionary response to such disarmament would therefore be similar to that to antibiotics, that is the evolution of resistance, and these virulence factors are thus a poor fit for the “disarm rather than kill” approach. However, the same bacteria also produce exotoxins (staphylococcal and streptococcal superantigens, e.g., SpeA). These too are virulence factors, because they harm the host, but they do so even in the absence of the microbe (Hennekinne, De Buyser, & Dragacci, 2012; Sriskandan, Unnikrishnan, Krausz, & Cohen, 1999). Their health effects thus have a component of direct damage that is not mediated by increased microbial growth and survival, and targeting these factors may conform better to the “disarm rather than kill” strategy.

The underlying problem is that the rationale for virulence factor inhibition is based on a distinction that the virulence factor framework does not make: the distinction between a factor that exerts a direct effect on host health independent of microbial load and one that harms the host indirectly by increasing pathogen density (consider the distinction by Allen et al., 2014 between virulence factors that are beneficial vs. nonbeneficial for the pathogen).

Due to the complexity of the mechanistic underpinnings, however, classifying factors according to these distinctions is not straight forward. For example, the health effect of the streptococcal pyrogenic exotoxin SpeA involves more than the direct harm discussed above; it also increases the density of streptococci in a model of nasopharyngeal infection (Kasper et al., 2014). And the antiphagocytic M protein, in addition to preventing bacterial killing, can induce vascular leakage and shock in the absence of the bacterium (Herwald et al., 2004). Both factors are thus associated with an increase in bacterial density, and thereby plausibly pathogen fitness, but they also exert direct damaging effects on the host, and these particular effects may or may not involve benefits for the microbe. Moreover, even when host harm and pathogen fitness are causally linked, the quantitative relationship between them may vary.

What is needed is therefore not a mere subdivision of virulence factors into different classes, but a framework in which we can partition and quantify the contribution made by these factors to different types of effects, that is the component of the effect on host health that is mediated by a change in bacterial load versus the component that is not. The practical goal of such an analysis would be to estimate the quantitative relationship between the host harm that can be prevented by inhibition of a given factor, and the strength of selection for resistance that inhibition would impose, that is a relationship between desired and undesired effects. Accordingly, a good target for virulence factor inhibition would be a factor that harms the host severely relative to its contribution to the fitness of the pathogen.

### 2.2 Molecular probiotics

In recent years, there has been growing interest in the beneficial effects of the microbiota (Young, 2017), and this has now begun to take a molecular turn (Allhorn, Arve, Brüggemann, & Lood, 2016; Mazmanian, Round, & Kasper, 2008; Wang et al., 2014) similar to that taken by the microbial pathogenesis field with the advancement of the virulence factor concept in the 1970s (Casadevall & Pirofski, 2001; Casadevall, 2009; Méthot & Alizon, 2014). This holds great promise, since just as harmful factors may be targeted with inhibitors or vaccines, so beneficial factors may be exploited as molecular probiotics to promote host health.

Continuing the analogy with virulence factors, however, beneficial factors may favour the host in different ways: by changing the density of the microbe, or by providing a health benefit at a given microbial density. The distinction is important, for if the factor is added exogenously, an effect on microbial density may have hard‐to‐foresee consequences for microbial ecology and evolution. And if the factor is used in the absence of the microbe, any effect that is mediated by a change in microbial density will be lost.

### 2.3 Virulence evolution

In evolutionary ecology, there is a strong tradition of mathematically modelling the evolution of pathogen virulence, and this body of theory can be used to predict the evolutionary consequences of medical interventions, such as vaccination (See Cressler, McLeod, Rozins, van den Hoogen, and Day (2016) for a recent review of the field and Gandon, Mackinnon, Nee, and Read (2001) for a medically relevant application.) It is striking, however, that the evolution of virulence is virtually never construed in terms of virulence factors. A likely reason is that the evolutionary models are built around assumptions about the relationship between host harm and different components of pathogen fitness, such as the rate of transmission and the duration of infection, whilst the virulence factor concept is centred on host harm, and does not take pathogen fitness into account (Casadevall & Pirofski, 2001, 2003; Casadevall, 2009).

However, components of pathogen fitness are closely related to the proliferation and density of the pathogen in the host (Acevedo et al., 2019; Fraser, Hollingsworth, Chapman, de Wolf, & Hanage, 2007; Råberg, 2012), and the relationship between host harm and pathogen density is therefore key to the study of virulence evolution (Leggett, Cornwallis, Buckling, & West, 2017). An analysis of virulence factors that decomposes their effects into two components—pathogen density versus the harm done relative to this density—would therefore facilitate their incorporation into a rich body of work in evolutionary biology.

## 3 THE HEALTH OF THE HOST AND THE EVOLUTION OF THE MICROBE

Summarizing thus far, the reason why concepts such as “virulence factor” are low on evolutionary implications is that they focus on the consequences for the host, whilst the evolutionary response to interventions depends on the effects on the microbe. The problem is largely conceptual, though, because studies of microbial molecules often assess their effects on both the microbe and the host, the latter as a measure of health and the former as microbial density. This points to a way forward, an analysis of microbial molecules that describes their effects in terms of two components: (i) the density of the microbe in the host and (ii) the relationship between host health and microbial density. Before going down that route, however, we set the stage by describing the two fundaments on which our framework is built: the health effects of microbial molecules, and the study of host resistance versus tolerance to infection.

### 3.1 The resistance–tolerance framework divides host defence into components with different evolutionary implications

Originally developed in plant biology (Caldwell, Schafer, Compton, & Patterson, 1958; Fineblum & Rausher, 1995; Simms, 2000), and later adopted by evolutionary animal ecology (Råberg et al., 2007, 2009), and more recently immunology (Medzhitov, Schneider, & Soares, 2012; Soares, Teixeira, & Moita, 2017), the resistance–tolerance framework partitions an organism's defence against infection (or any type of attack) into two components: *resistance* is the host's ability to limit the density of the infecting microbe, and *tolerance* is its ability to cope with a given microbial density. More formally, the difference between resistance and tolerance can be illustrated with a linear regression of the form *Health = a + bc.* Here, *c* (colony forming units; cfu) is the density of the microbe in the host, and its average value for a given type of host is thus a measure of the resistance of that host type. The effect of changing the density is estimated by *b* (the slope), which is then the tolerance, a shallow slope meaning high tolerance. And *a*, lastly, is the baseline health of uninfected hosts, often referred to as “vigour.” This analysis has been applied to a number of host‐pathogen systems and shed considerable light on the genetics and physiology of host defence (Hayward et al., 2014; Jamieson et al., 2013; Regoes et al., 2014; Sahoo, Del Barrio, Miller, & Re, 2014; Soares et al., 2017; Troha, Im, Revah, Lazzaro, & Buchon, 2018; Wang et al., 2016).

**Figure 1:**
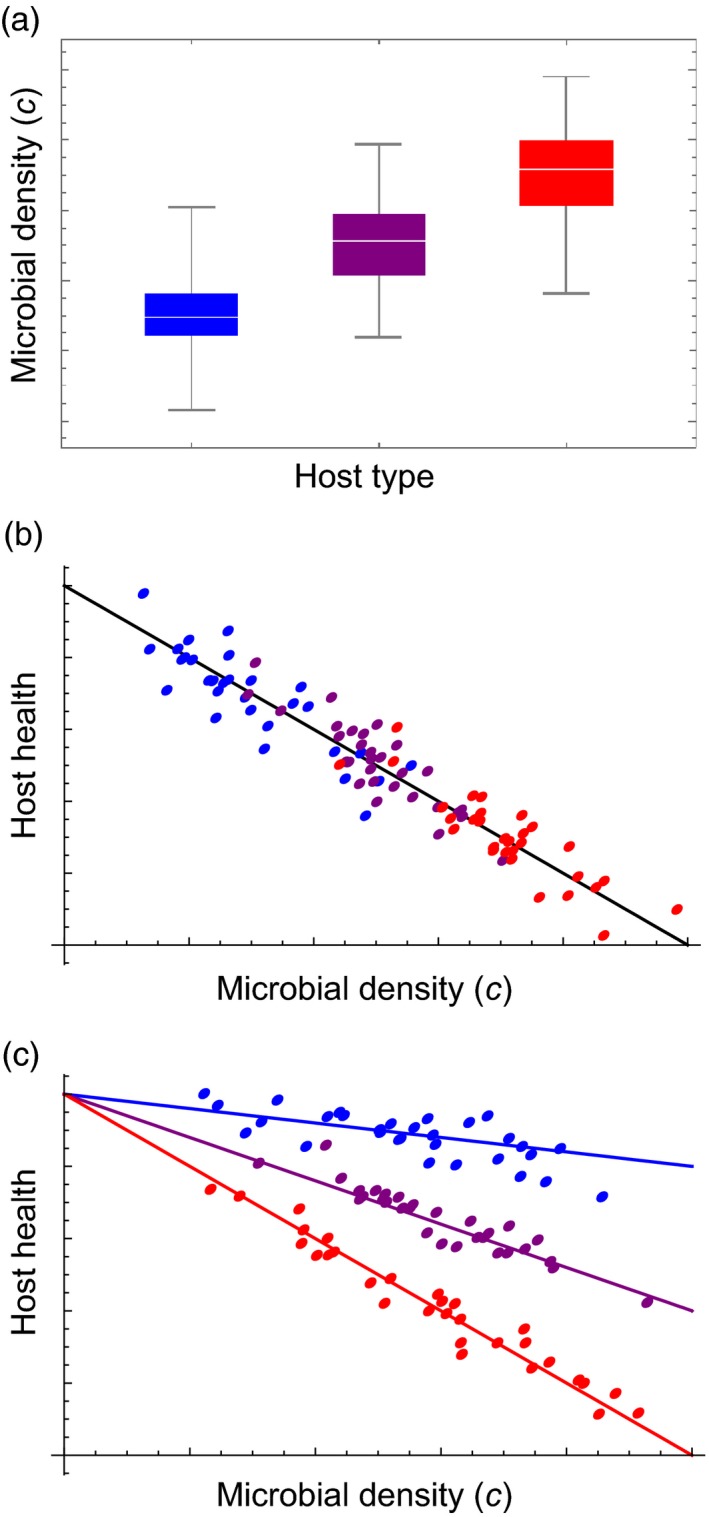
(a) The microbial densities for three host types (blue, purple, red) are illustrated. The host types vary in resistance. (b) The same simulated data as in (a) are plotted with host health against microbial density. The host types (blue, purple, red; as in [a]) vary in resistance, but not in tolerance. (c) The health–density relationships for three host types (blue, purple, red; not identical to those in [a] and [b]) are illustrated. Host types vary in tolerance, but not in resistance

To quantify the extent to which the variation in health outcome among hosts is due to variation in resistance and tolerance, respectively, a range of host types (e.g., different genotypes) are infected with a single type of pathogen. The data are then analysed for variation among host types in their average microbial density ( $\bar{c}$ ) and in their slopes of health on density (*b*). Illustrating this, Figure 1a shows the microbial density for three different host types. These vary in resistance, but as seen in Figure 1b, they are similar in tolerance. Figure 1c, in contrast, shows the health–density relationships for three host types with similar resistance, but varying tolerance. Box 1 provides details on the analyses.

The rationale for this partitioning of variation in defence into two components lies in the evolutionary implications. Host resistance is detrimental to the microbe and is therefore expected to select for microbial counter‐adaptations, in much the same way as antibiotic treatment does. Tolerance, in contrast, modulating host health at a given microbial density, does not directly harm the microbe and should therefore not provoke this sort of evolutionary response (Best, White, & Boots, 2014; Rausher, 2001), but see Vale, Fenton, and Brown (2014) for a critical discussion of the evolutionary implications of targeting tolerance.

Though developed for evolutionary analysis, the resistance–tolerance framework chimes well with medical practice. Consider for example the treatment of sepsis, a leading cause of death (Deutschman & Tracey, 2014). This includes antibiotics, which augment host resistance by pushing down the density of the pathogen, but also fluid therapy to compensate for vascular leakage induced by the immune response, and thereby attenuate the damage done at a given pathogen density (Deutschman & Tracey, 2014), thus enhancing tolerance.

### 3.2 Microbial molecules are crucial to health outcome

Though influenced by host defence and medical interventions, the consequences of host–microbe interactions for host health also critically depend on properties of the microbe, as evidenced by the differences in health outcome for infection and colonization with microbes of different strains and species. The mechanistic basis for these properties has therefore been subject to intense research (Carruthers, Cotter, & Kumamoto, 2007; Fittipaldi, Segura, Grenier, & Gottschalk, 2012; Holt & Bramanti, 1991; van Sorge et al., 2014), and as a result, a large number of microbial molecules have been identified that contribute to host harm during infection (Falkow, 1988; Kao, Sheu, & Wu, 2016; Liu, 2009; Patenge, Fiedler, & Kreikemeyer, 2013). These molecules—the *virulence factors*—are central to our understanding of microbial pathogenesis, and “virulence factor” is arguably the most important concept in the field. The positive effects that microbes exert on host health have been studied to a lesser extent, but here too specific molecules mediating the effects have been found (Allhorn et al., 2016; Christensen & Brüggemann, 2014; Lai et al., 2009; Mazmanian et al., 2008; Schommer & Gallo, 2013).

The critical role that such molecules play in shaping the outcome of host–microbe interactions is well illustrated by the health effects of different strains of *E. coli*. These range from the benefits of vitamin K production (Kindberg, Suttie, Uchida, Hirauchi, & Nakao, 1987; Resta, 2009) to the severe harm of the haemolytic uraemic syndrome caused by the EHEC shiga toxin (Fakhouri, Zuber, Frémeaux‐Bacchi, & Loirat, 2017). On a more general note, microbial strains vary in what virulence factors they encode (Bosi et al., 2016; D’Auria, Jiménez‐Hernández, Peris‐Bondia, Moya, & Latorre, 2010) and how these factors are expressed (Jhingan et al., 2016), and this forms a mechanistic basis for the variation in the host harm that, even conspecific, microbes incur.

## 4 PROLIFERATION AND BENEVOLENCE—THE MICROBIAL COUNTERPARTS OF RESISTANCE AND TOLERANCE

Having appraised the analytic approach of the resistance–tolerance field and the health effects of microbial molecules, we now apply the former to the latter. We begin with a consideration of microbial genetic variation, since this is most closely analogous to the resistance–tolerance analysis of genetic variation in the host, and then move on to gene knockouts and the corresponding effector molecules.

### 4.1 Genetic variation and the properties of hosts and microbes

**Figure 2:**
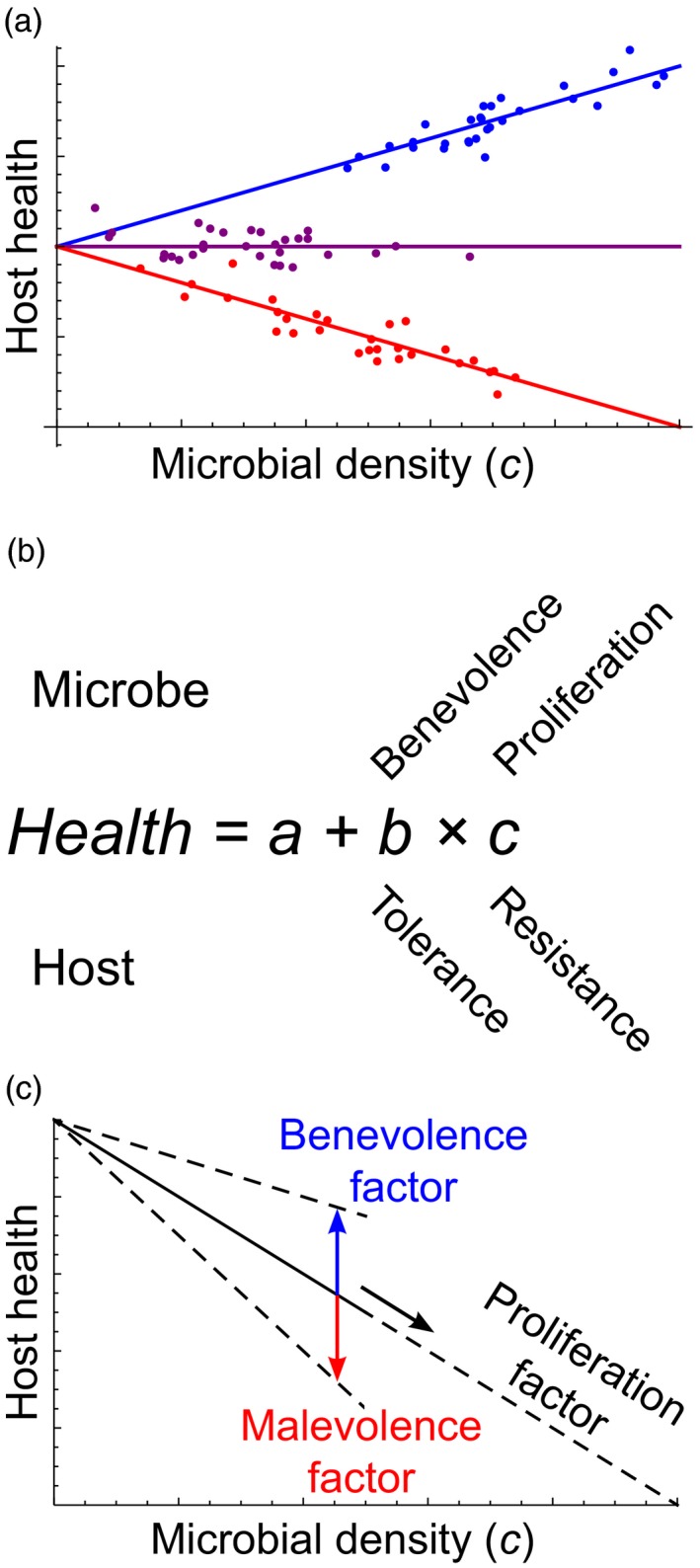
(a) Three host–microbe combinations are represented. The health effects are positive (*b > 0*; blue), neutral (*b = 0*; purple), and negative (*b < 0*; red). There is also variation in mean microbial density (*c̄*). (b) Host health is represented as baseline health (*a*) plus the effect of the host‐microbe interaction (*bc*). The contributions of the microbe and host are given at the top and bottom, respectively. (c) The solid line represents the health–density relationship for a knockout strain, and the dashed lines represent possible health–density relationships for the corresponding wild type. If the wild type has a more positive or less negative slope than the knockout (*b_wild_type_ > b_knockout_*), the factor is a benevolence factor (blue arrow). If instead the wild type has a less positive or more negative slope than the knockout (*b_wild_type_ < b_knockout_*), the factor is a malevolence factor (red arrow). If the wild type attains higher densities than the knockout ( ${\bar{c}}_{wild\_type}>{\bar{c}}_{\mathrm{knockout}}$ ), the factor is a proliferation factor (black arrow). A pure benevolence or malevolence factor changes only the slope, not the average density, and a pure proliferation factor increases the density without affecting the slope. A single factor may affect both benevolence and proliferation

Figure 2a shows the relationship between host health and microbial density for three host–microbe combinations. As described above, experiments designed to generate this sort of data form the basis of resistance–tolerance research. In such experiments, a single genotype of the microbe is inoculated into different genotypes of the host, and the results shown in Figure 2a would indicate that host types vary in both tolerance (slope) and resistance (average density).

However, the experiment can also be designed the other way around, with a single type of host infected by different microbial strains, and the variation in slope (*b*) and average microbial density ( $\bar{c}$ ) shown in Figure 2a would then be due to variation in the microbe. The microbial properties to which this variation pertains we call *benevolence* (*b*) and *proliferation* ( $\bar{c}$ ), and as illustrated in Figure 2b, these are analogous to the host properties of tolerance and resistance, respectively. That is, resistance and proliferation both refer to the average microbial density ( $\bar{c}$ ), and tolerance and benevolence to the slope (*b*). The difference is that variation in resistance and tolerance is due to variation in the host, whereas variation in proliferation and benevolence is due to variation in the microbe.

Benevolence thus represents the health effect that a microbe exerts on a host relative to its density. We use benevolence to denote the property as such, despite its positive connotations (confer the analogy with tolerance), but contrast it with *malevolence* when referring to specified instances where the direction of the health effect is given. Accordingly, we call a microbe *benevolent*, precisely if it benefits the host (*b > 0*) within the relevant range of densities, and *malevolent*, if it is detrimental (*b < 0*). To cause disease, a pathogen must be malevolent, and the product of its malevolence and proliferation ( $b\bar{c}$ ) is the virulence.

This distinction between benevolence, on the one hand, and proliferation, on the other, addresses the problem with antivirulence therapeutics discussed above. The appropriate target for the “disarm rather than kill” approach is not virulence ( $b\bar{c}$ ), because this incorporates microbial density ( $\bar{c}$ ), and thereby the inhibition and killing of the microbe. The target is benevolence (*b*), the health effect relative to the density. Modulating benevolence is expected to induce less selection for counter‐adaptations (such as drug resistance), because, in its pure form, it leaves the density of the microbe, and thereby a key component of its Darwinian fitness, unaltered.

But as promising as this may seem, it also highlights two important problems. Firstly, if benevolence and proliferation are mechanistically linked, it may be impossible to decouple host harm from pathogen fitness, and thereby selection on resistance. And secondly, even if it were possible to limit host damage without decreasing pathogen load, this may still alter the pathogen's fitness landscape by affecting transmission among hosts, potentially resulting in the evolution of both drug resistance and increased virulence (Vale et al., 2014). In current virulence evolution theory, the canonical way for damage limitation to affect transmission is by decreasing host mortality, and thereby increase the duration of transmission. A drug that reduces mortality, even if purely via malevolence, would thus still affect the pathogen's fitness and could thereby provoke an evolutionary response. On the other hand, antivirulence therapeutics may be best suited for mild infections (Wollein Waldetoft & Brown, 2017), where host mortality is not an important evolutionary force. And conversely, conditions with substantive mortality, often involving infection of normally sterile sites, plausibly require that the proliferation component of virulence be targeted, and the pathogen cleared. In any case, it is important to keep in mind that whilst microbial density (proliferation) is related to microbial fitness, it does not capture all its aspects.

Box 1 details the study designs and associated data analyses and extends them to include joint effects of variation in both microbe and host, as well as health outcomes with non‐normally distributed data (e.g., survival) and nonlinear relationships between host health and microbial density.

### 4.2 Genetic manipulation and the effects of specific molecules

To translate benevolence and proliferation into evolutionarily informed biomedical interventions, we need to move beyond the analysis of uncharacterized genetic variation (i.e., variation among microbial strains as outlined above) and identify the specific effector molecules responsible. To this end, we turn to the experimental tradition of molecular microbial pathogenesis. Guided by the molecular Koch's postulates, this field studies microbial molecules by knocking out the genes that encode them. The knockout strains are then compared to their isogenic wild type and complemented control strains in experimental infections. Typical readouts include measures of host health and microbial density, a negative effect on health qualifying the molecule as a virulence factor.

Given information on health and density, we can divide the effect of the molecule into benevolence and proliferation. An effect on benevolence amounts to a change in the slope of host health on microbial density (*b*). If the molecule increases the slope, making it more positive or less negative (*b_wild_type_ > b_knockout_*), we call it a *benevolence factor*, and if the opposite is the case (*b_wild_type_ < b_knockout_*), it is a *malevolence factor*. Similarly, a molecule that increases the average density of the microbe ( ${\bar{c}}_{wild\_type}>{\bar{c}}_{\mathrm{knockout}}$ ) is a *proliferation factor* (Figure 2c).

To minimize selection for drug resistance, a candidate target molecule for antivirulence therapeutics should exert its effect primarily via malevolence rather than proliferation. And similarly, a molecular probiotic should have a large component of benevolence relative to proliferation, since the effect on proliferation will be lost in the absence of the microbe, and in its presence may interfere with microbial ecology and evolution.

Experimental designs, statistical analyses, and extensions to include host factors, non‐normally distributed data, and nonlinear relationships are described in Box 1.

## 5 EVIDENCE FOR PROLIFERATION AND BENEVOLENCE

### 5.1 Genetic variation among strains

The effects that microbes and their molecules exert on host health are not normally analysed along these lines, but some preliminary conclusions can nonetheless be drawn. Strains of a microbe often differ in the densities they attain in a given host, that is, they vary in proliferation (Johnson et al., 1998). There are also studies showing that microbial density and effects on host health, as measured by for example survival rates, are not well correlated across strains (Wang et al., 2016), which indicates that strains vary in benevolence. However, to date, very few studies have explicitly tested for variation in benevolence among strains. A recent exception is a study of HIV‐1, which demonstrated significant genetic variation in malevolence using a phylogenetic approach (Baeten et al., 2007; Bertels et al., 2018).

Contributing to this relative lack of studies of benevolence may be the fact that the trade‐off theory of virulence evolution is focused on variation in the proliferation component of virulence, whilst the benevolence component is assumed constant, as part of the trade‐off. If variation in benevolence were to be consistently found in empirical studies, this would therefore pose an interesting problem for virulence evolution research.

### 5.2 Specific molecular factors

A number of studies report that the presence of a factor increases the density of a microbe in experimental hosts, as determined by colony counts for various body sites (Belda et al., 2012; Crotty Alexander et al., 2010; Kasper et al., 2014), that is, they identify proliferation factors.

Specific molecules affecting benevolence have also been reported. For example, Yoong and Pier (2012) used a mouse pneumonia model to investigate the effect of Panton‐Valentine leukocidin (PVL) produced by many strains of methicillin resistant *Staphylococcus aureus* and found that mice infected with wild‐type strains had lower mortality than those infected with isogenic ∆*pvl* strains, despite there being no corresponding difference in cfu counts in the lungs. PVL thus improved host health relative to microbial density, which makes it a strong candidate for being a benevolence factor under the particular conditions studied. Similarly, Kolar et al. (2015) found that mice infected with wild‐type group B streptococci showed lower mortality despite higher microbial densities than mice infected with an isogenic knockout strain lacking a hyaluronidase. The results were corroborated by investigation of the effect of purified hyaluronidase on LPS‐induced acute lung injury. In both studies, immunomodulation was implicated in the benevolent effect.

In contrast to the studies above, Louie, Song, Hotson, Thomas Tate, and Schneider (2016), explicitly addressing health–density relationships in a *Listeria‐Drosophila* infection model, found that the actin assembly‐inducing protein (a known virulence factor) changed this relationship to the detriment of the host, thus making it a strong candidate for being a malevolence factor. Moreover, Burnside et al. (2010) infected mice with *Staphylococcus aureus* having or lacking the serine/threonine phosphatase Stp1 and found that the presence of Stp1 increased mortality but not pathogen load. This indicates that Stp1 is a malevolence factor in that system. The study is particularly interesting, because it highlights the complexity of infection biology and the need to integrate evolutionary and mechanistic thinking. For whilst the malevolent nature of Stp1 would make it evolutionarily suitable for antivirulence therapeutics, its intracellular location makes it difficult to target, a problem that may, in turn, be solved by the suggestion in the study that the health effect of Stp1 is mediated by an extracellular toxin.

In summary, there are a number of studies that identify what are plausibly benevolence and malevolence factors, but firm conclusions and the quantification of the effect on benevolence would require the statistical approach outlined in Box 1.

## 6 CONCLUDING REMARKS

Here, we have proposed a conceptual framework for host–microbe interactions, described the corresponding data analysis, and reviewed existing evidence for the types of effects that the framework identifies. These effects—proliferation and benevolence—are analogous to resistance and tolerance, but whilst the latter pertain to the host, the former are traits of the infecting microbe, conferred by the molecules that current studies characterize. As such, the framework can be used to identify candidate molecules for exploitation and intervention, and it does so in a way that facilitates their incorporation into existing work in evolutionary biology to predict the potential evolutionary responses to medical applications.

In conclusion, the proliferation–benevolence framework provides a common ground for mechanistic and evolutionary approaches and begins to lay a foundation for work on complex problems that require integrated contributions from both lines of research.

## CONFLICT OF INTEREST

None declared.

## Data Availability

There are no data associated with this paper.
